# Investigating the Gut Microbiota Profile in Prehypertensive Individuals Exhibiting Phlegm-Dampness Constitution

**DOI:** 10.3389/fcimb.2025.1507076

**Published:** 2025-03-04

**Authors:** Ning Yu, Yaotang Yang, Guangyun Wang, Yanhong Wang, Mei Feng, Peilin Yang, Shuang Liu, Rui-rui Wang, Lei Zhang

**Affiliations:** ^1^ School of Public Health, Shanghai Innovation Center of Traditional Chinese Medicine Health Service, Shanghai University of Traditional Chinese Medicine, Shanghai, China; ^2^ State Key Laboratory of Integration and Innovation of Classic Formula and Modern Chinese Medicine, Shanghai University of Traditional Chinese Medicine, Shanghai, China; ^3^ College of Sport and Health, Shandong Sport University, Jinan, China; ^4^ Centre for Health Management, Rizhao hospital of Traditional Chinese Medicine, Rizhao, China; ^5^ Department of Cardiology, Rizhao hospital of Traditional Chinese Medicine, Rizhao, China; ^6^ Shanxi Institute for Function Food, Shanxi Agricultural University, Taiyuan, China

**Keywords:** prehypertension, phlegm dampness constitution, gut microbiota, biomarker, 16S rRNA

## Abstract

**Background:**

Prehypertension is the preclinical stage of hypertension, which is more likely to develop into hypertension than normal blood pressure. Although the body may experience pathological changes at this stage, there are often no symptoms. Chinese medicine constitution theory is widely used to assess an individual’s health and disease status, which provides a new method for disease prevention. The phlegm-dampness constitution (PDC) is the main constitution in prehypertension. Dysbiosis of the intestinal flora is considered to be related to the development of hypertension. However, the characteristics of the intestinal flora in prehypertensive populations with PDCs are still unknown.

**Methods:**

16S rRNA gene sequencing of fecal samples was performed in this study, which included 30 prehypertensive subjects with PDCs, 30 nonphlegm-dampness constitution (NPDC) prehypertensive individuals with balanced constitution, and 30 ideal blood pressure subjects with balanced constitution (BC). On the basis of the composition of the intestinal flora, a random forest classifier was constructed to screen the specific bacteria of the prehypertensive PDC population, and the diagnostic efficiency was determined by the area under the curve (AUC).

**Results:**

At the phylum level, the abundance of *Bacteroidetes* decreased in the PDC group compared with the NPDC group. *Bacteroides* was the most important genus at the genus level. Compared with those in the NPDC or BC group, the relative abundances of *o_RF39*, *f_Porphyromonadaceae*, *f_Christensenellaceae*, *g_parabacteroides*, and *g_nitrobacteria* in the PDC group were significantly greater. The random forest analysis results revealed that *Alistipes*, *Butyricimonas*, *Odoribacter*, *Parabacteroides*, and *Corynebacterium* are bacterial genera that significantly differ between the PDC and NPDC groups and greatly contribute to group differentiation. Receiver operating characteristic (ROC) analysis revealed that the AUC range of differential bacteria and its combined diagnostic model ranged from 0.653 (95% CI: 0.511–0.794) to 0.706 (95% CI: 0.573–0.838), suggesting that it is a potential risk marker for phlegm-dampness constitution with prehypertension.

**Conclusions:**

Our study indicates that PDC individuals with prehypertension can be distinguished from NPDC individuals according to their gut microbiome characteristics. Prevention and treatment measures based on these biomarkers may be beneficial in opening new ideas and directions for identifying more aggressive and effective interventions for prehypertensive populations.

## 1 Introduction

Hypertension is a common chronic disease, a major risk factor for cardiovascular and cerebrovascular diseases (Lawes et al., 2003), and one of the most important challenges for public health, especially in developing countries (Rapsomaniki et al., 2014). Prehypertension is the stage between ideal blood pressure and hypertension, and the risk of hypertension and cardiovascular disease is significantly greater than that of the population with normal blood pressure (Brunström and Carlberg, 2018). However, there are often no symptoms at this stage, even if the body has undergone pathological changes that are not clinically recognized, which hinders the resolution of the problem (Fuchs and Whelton, 2020).

Traditional Chinese medicine (TCM) constitution is a relatively stable internal characteristic that is based on innate endowment and acquired factors and combines the morphological structure, physiological function, and pathological changes of the body (Balouchi et al., 2022). On the basis of its classification and judgment criteria, the population can be divided into nine categories. The TCM constitution is applied to evaluate the health and disease status of individuals, especially in the study of various metabolic diseases and in the management of public health (Qi et al., 2018), which compensates for defects in diagnosis and highlights a new direction for health and disease assessment (Jin and Chen, 2024). An unbalanced constitution indicates that the body may be susceptible to disease (Vasan et al., 2001). In addition to balanced constitutions, which are relatively healthy, the other constitutions are unbalanced. The constitution of phlegm dampness constitution is due to dysfunctional water−liquid metabolism and the accumulation of phlegm dampness in the body (Wang et al., 2006). PDCs are more prone to dyslipidemia (Deng et al., 2013) and are believed to underlie the pathogenesis of insulin resistance (Yang et al., 2012), which is also closely associated with overweight and obesity (Li et al., 2017; Shin et al., 2022) as well as diabetes mellitus, metabolic syndrome (MS), and hypertension (Zhu et al., 2010; Han et al., 2013; Liang et al., 2017; Fang et al., 2021; Cao and Hou, 2023). Compared with other constitutions, PDC has a significant metabolic disorder trend (Tao et al., 2024). A meta-analysis that included 17 studies involving 8118 participants indicated that PDC is considered to be the most predominant abnormal constitution of prehypertension (Mu et al., 2023).

According to the theory of TCM, the intestinal tract belongs to the spleen-gastric system. Researchers have concluded that the intestinal flora is inextricably linked to the spleen system (Guo et al., 2021) and that spleen dysfunction is the basis and key link in the development of metabolic disorders in PDC (Zhang et al., 2013; Wu et al., 2022). There is a close relationship between intestinal microorganisms, PDC and metabolic diseases. Gut microbes play important roles in maintaining the physiological homeostasis of the body and are recognized as important intersections in the study of PDC and metabolic disorders (Liang et al., 2020). Studies have shown that an imbalance in the intestinal flora is related to changes in host blood pressure (Yang et al., 2015). J. Li et al. (Li et al., 2017) revealed that the abundance and diversity of gut microbes were significantly reduced in prehypertensive and hypertensive populations. Y. Zou et al. (Zou et al., 2022) reported that dysbiosis of the gut fungal flora had already been observed in the prehypertensive stage. However, the characteristics of the intestinal flora in prehypertensive individuals with PDC are still unclear.

Therefore, we speculate that prehypertensive populations with PDCs may have different microbial compositions and structures than those with NPDCs. In this study, we utilized high-throughput sequencing technology to investigate the characteristics and diagnostic efficacy of the intestinal flora in prehypertensive populations with phlegm dampness to identify positive and effective new perspectives and directions for the prevention and control of hypertension and prehypertension.

## 2 Materials and methods

### 2.1 Research subjects

We recruited participants from the physical examination population of Shandong University of Traditional Chinese Medicine affiliated with Rizhao Hospital of TCM from April 2021 to December 2021 and divided them into 3 groups with 30 participants in each group. PDC and NPDC subjects with prehypertension and BC subjects with ideal blood pressure. All the subjects met the criteria for phlegm dampness or balanced constitution (ZZYXH/T157-2009) (China Association of Chinese Medicine, 2009), diagnostic criteria for prehypertension and ideal blood pressure, according to the Chinese Guidelines for Prevention and Treatment of Hypertension (Joint Committee for Guideline Revision, 2019). Patients with essential hypertension and/or secondary hypertension; individuals with severe diseases of the liver, kidney, lung, brain, endocrine, hematopoietic, or other organ systems; pregnant or pregnant or lactating women; those who received other treatments that may have affected the population observed in this study; and those who also received probiotics and other intestinal microecological preparations were excluded. All participants were not allowed to take any of the following drugs: antibiotics, laxatives, antidepressants, probiotic supplements, PPI preparations, bismuth, hormones, or chemotherapy drugs at least 4 weeks before the study began. The study scheme has passed the ethical review of the China Registered Clinical Trial Ethics Committee (ethics review document number: ChiECRCT20210464), and all participants are required to provide informed consent.

### 2.2 Sample collection

All clinical information was collected according to standard procedures. Peripheral venous blood was drawn after the participants were enrolled in the study, and the participants were provided with a stool sampler and detailed graphic instructions for sample collection. Freshly collected stool samples from each participant were frozen overnight in liquid nitrogen and stored in a -80°C freezer. After collection, all the samples were sent to Shanghai Personalbio Biotechnology Co., Ltd., for high-throughput sequencing.

### 2.3 Sample pretreatment

The fecal sample was removed from the refrigerator and added to a centrifuge tube containing the extracted lysate for grinding at a frequency of 60 Hz (Shanghai Jingxin Multisample Tissue grinder, Model: Tissuelyser-48).

### 2.4 Extraction of total DNA from the intestinal flora

For the pretreated samples, nucleic acid was extracted via the OMEGA Soil DNA Kit (D5635-02) (Omega Bio-Tek, Norcross, GA, USA). The molecular size was determined via 0.8% agarose gel electrophoresis, and the DNA was quantified via a Nanodrop. Extraction was performed via OMEGA’s Mag-Bind Soil DNA Kit (catalog number: M5635-02).

### 2.5 PCR amplification of intestinal flora DNA

The highly variable V_3_V_4_ region of the bacterial 16S rRNA gene, with a length of approximately 480 bp, was selected for PCR amplification. The sequences of the primers used were 338F (5’- barcode+ACTCCTACGGGAGGCAGCA-3’) and 806R (5’-GGACTACHVGGGTWTCTAAT-3’). The barcode in the preprimer is a 7–10-base oligonucleotide sequence used to distinguish different samples in the same library. NEB Q5 DNA high-fidelity polymerase was used for PCR. After the components required for the PCR were configured, the template DNA was predenatured at 98°C for 5 minutes on the PCR apparatus to fully denature and then entered the amplification cycle. During each cycle, the template is denatured by holding for 30 s before 98°C, and then the temperature is lowered to 53°C for 30 s so that the primer and the template are fully annealed. At 72°C for 45 s, the primer was extended on the template, the DNA was synthesized, and a cycle was completed. This cycle was repeated 25 times to enrich the amplified DNA fragments. Finally, the product was kept at 72°C for 5 min so that the extension of the product was complete, and the product was preserved at 12°C. The products were amplified via 2% agarose gel electrophoresis, and the target fragments were cut and then recovered with an Axygen gel recovery kit. The PCR products were quantified on a microplate reader (Bio Tek, FLx800) with a Quant-iT Pico Green ds DNA Assay Kit, after which the samples were mixed according to the amount of data required for each sample.

### 2.6 Library construction, quality inspection, and sequencing

The Illumina’s Tru Seq Nano DNA LT Library Prep Kit was used for library construction, library quality, and quantification. A 1 μL library was collected, and a 2100 quality inspection of the library was conducted with an Agilent High Sensitivity DNA Kit on an Agilent Bioanalyzer machine. The qualified library should have a single peak and no joint peak. A Quant-iT PicoGreen dsDNA Assay Kit was used to quantify the library on a Promega QuantiFluor, and the concentrations of the qualified libraries were above 2 nM after calculation. Qualified libraries were sequenced to 2×250 bp in length via the NovaSeq 6000 SP Reagent Kit (500 cycles) on the Illumina NovaSeq machine.

### 2.7 Data quality control and analysis

QIIME2 2019.4 was used to analyze microbiome biology information (https://docs.qiime2.org/2019.4/tutorials/).

#### 2.7.1 ASV classification and classification status identification

A certain number of sequences were randomly selected from each sample to reach a uniform depth to predict the ASVs and the relative abundance of each sample at this sequencing depth. The extraction depth was 95% of the minimum sample sequence quantity. Using the Greengenes database, the characteristic sequences of ASVs were compared with reference sequences in the database to obtain taxonomic information corresponding to each ASV. The ASVs with only 1 sequence were removed, and the abundance matrix with rare ASVs removed was used in a subsequent series of analyses. Moreover, R software was used to draw the identification results of each sample at each classification level into a bar chart to visually compare the differences in the number of ASVs and classification status identification results of different samples.

#### 2.7.2 Analysis of α diversity and β diversity

The α diversity index and β diversity index represent within-habitat diversity and between-habitat diversity, respectively (Kemp and Aller, 2004). QIIME2 software was used to randomly sample the total number of sequences in the ASV abundance matrix at different depths, and rarefaction curves were drawn to judge whether they could reflect the microbial diversity contained in the samples. Diversity indices such as Chao1, observed species, Shannon and Simpson indices, Faith’s PD, and Good’s coverage were calculated and compared to evaluate sample diversity. The Kruskal−Wallis rank sum test was used as a *post hoc* test to verify the significance of the differences. The multidimensional microbial data were reduced via principal coordinate analysis (PCoA) and represented by the difference in distance between samples. The community differences among the samples were characterized by the Bray−Curtis distance. We applied the following analysis software to complete the analysis: QIIME2 (2019.4), R language, and the ape package. Statistical analysis of similarities (ANOSIM) was used to examine the significance of differences in microbial community structure among the groups.

#### 2.7.3 Taxonomic composition analysis

According to the results of ASV classification and taxonomic status identification, QIIME2 software was used to obtain the species composition and abundance table of each sample at different taxonomic levels of phyla, classes, orders, families, genera, and species, and the analysis results are presented in a bar chart.

#### 2.7.4 Differential analysis among taxonomic groups

The linear discriminant analysis effect size (LEfSe) method, which combines nonparametric Kruskal−Wallis and Wilcoxon rank sum tests with linear discriminant analysis (LDA), was used to detect taxa with rich differences among each group. The results are shown in the LDA value distribution histogram and taxonomic branch diagram. The former shows the species significantly enriched in each group and their importance, whereas the latter shows the taxonomic hierarchy of the marker species in each group of samples. Through analysis software, the Python LEfSe package, R language, and ggtree package were used.

### 2.8 Diagnostic model construction

To evaluate the diagnostic effect on intestinal microorganisms, we determined the number of optimal genera through the analysis of the tenfold cross-validation error rate curve, constructed a random forest model (RFM), combined it with the screening results of LEfSe analysis (LDA>2), and calculated the AUC value of the bacteria that jointly met the above conditions. ROC curves were generated to evaluate the efficacy of the diagnostic model for gut microbes. The confidence intervals (CIs) of the area under the curve (AUC) and receiver operating characteristic (ROC) curves were calculated with GraphPad Prism (v 10.0, San Diego, California, CA, USA).

### 2.9 Statistical analysis

One-way analysis of variance (ANOVA) was applied for continuous variables with a normal distribution and homogeneity of variance; otherwise, a nonparametric test was used. Multiple comparisons were performed via the least significant difference (LSD) t test. Continuous variables are expressed as the mean ± standard deviation (SD) or median and quartile spacing (P25, P75). Categorical variables were compared via the χ^2^ test and are expressed as numbers (percentages). Statistical analysis was performed via GraphPad Prism (10.0, San Diego, California, CA, USA) and SPSS 26.0 (IBM, Armonk, New York), and *P* < 0.05 was considered statistically significant. Relative abundance differences in flora were analyzed for correlations between clinical indicators and the Personalbio gene cloud platform (https://www.genescloud.cn/).

## 3 Results

### 3.1 Clinical features

In addition to blood pressure, individuals in the PDC and NPDC groups presented greater body weight, BMI, body fat mass, and waist circumference (WC) than those in the BC group did. Among them, the differences in WC and BMI were statistically significant in both the NPDC and PDC groups compared with the BC group (*P* < 0.0001). In addition, there were no statistically significant differences in sex or age distribution among the three groups. Although the weight, WC, BMI, body fat, and TC in the PDC group were greater than those in the NPDC group were, the differences were not statistically significant. In a sense, distinguishing PDC and NPDC individuals with prehypertension on the basis of only obesity, glucose, and lipid metabolism indicators was difficult (**Table 1**).

**Table 1:** Baseline characteristics of the participants.

Variable	BC (n=30)	NPDC (n=30)	PDC (n=30)	*P* value
Age (y)	49.00 (25.50-56.50)	51.00 (33.00-61.00)	41.00 (31.25-51.25)	0.200
Gender (male %)	9 (30.0)	13 (43.3)	14 (46.7)	0.378
Height (cm)	164.40 (161.90-169.70)	164.60 (161.20-174.40)	168.05 (160.75-174.73)	0.526
Weight (kg)	62.70 (57.30-68.75)	67.70 (63.30-80.00)	71.90 (67.05-82.65)^*^	<0.0001
BMI (kg/m^2^)	23.52 ± 3.07	25.66 ± 3.06^*^	26.47 ± 2.74^*^	0.001
WC (mm)	80.17 ± 9.03	89.17 ± 9.59^*^	89.97 ± 8.84^*^	<0.0001
Body fat (%)	28.42 ± 4.70	30.00 ± 5.41	30.56 ± 5.40	0.259
SBP (mmHg)	110.00 (101.5 -115.50)	131.00 (123.00-138.00)^*^	124.50 (121.25-131.00)^*^	<0.0001
DBP (mmHg)	70.00 (64.50-74.00)	84.00 (81.00-88.00)^*^	78.00 (72.75-86.25)^*^	<0.0001
HR (beats/min)	71.67 ± 8.85	73.43 ± 9.64	75.07 ± 9.52	0.389
TC (mmol/l)	5.11 ± 1.02	5.40 ± 0.98	5.22 ± 1.09	0.566
TG (mmol/l)	0.87 (0.61 -1.49)	1.07 (0.92 -1.58)	1.27 (0.66-2.31)	0.198
L-DLC (mmol/l)	2.79 (2.22-3.26)	3.10 (2.54-3.71)	2.72 (2.29-3.27)	0.254
H-DLC (mmol/l)	1.66 ± 0.30	1.60 ± 0.47	1.51 ± 0.37	0.315
Lp (a)	144.00 (58.00-238.50)	136.00 (46.00-364.00)	102.00 (40.00-158.50)	0.458
FBG (mmol/l)	4.50 (4.30 - 4.80)	4.80 (4.30 -5.20)	4.75 (4.40-5.03)	0.059
BUN (mmol/l)	4.30 (3.50-5.15)	4.40 (3.70-5.10)	4.90 (4.08-5.83)	0.329
CR (μmol/l)	61.00 (54.50-68.50)	64.00 (54.00-73.00)	65.50 (59.00-78.00)	0.111
UA (ng/dL)	292.00 (251.00-342.00)	320.00 (268.00-387.00)	323.00 (248.00-394.50)	0.509

The data are presented as the means ± SDs, medians (P25–P75) or numbers (%). Compared with the BC group, ^*^ *P* < 0.05; compared with the NPDC group, ^#^ *P* < 0.05; No significant difference between PDC and NPDC groups. BMI, body mass index; WC, waist circumference; SBP, systolic blood pressure; DBP, diastolic blood pressure; HR, heart rate; TC, total cholesterol; TG, triglycerides; LDL-C, low-density lipoprotein cholesterol; HDL-C, high-density lipoprotein cholesterol; apoA, apoprotein A; FBG, fasting blood glucose; BUN, blood urea nitrogen; CR, creatinine; UA, uric acid.

### 3.2 Comparison of intestinal microbial diversity among the NPDC, PDC, and BC populations

**Figure 1:**
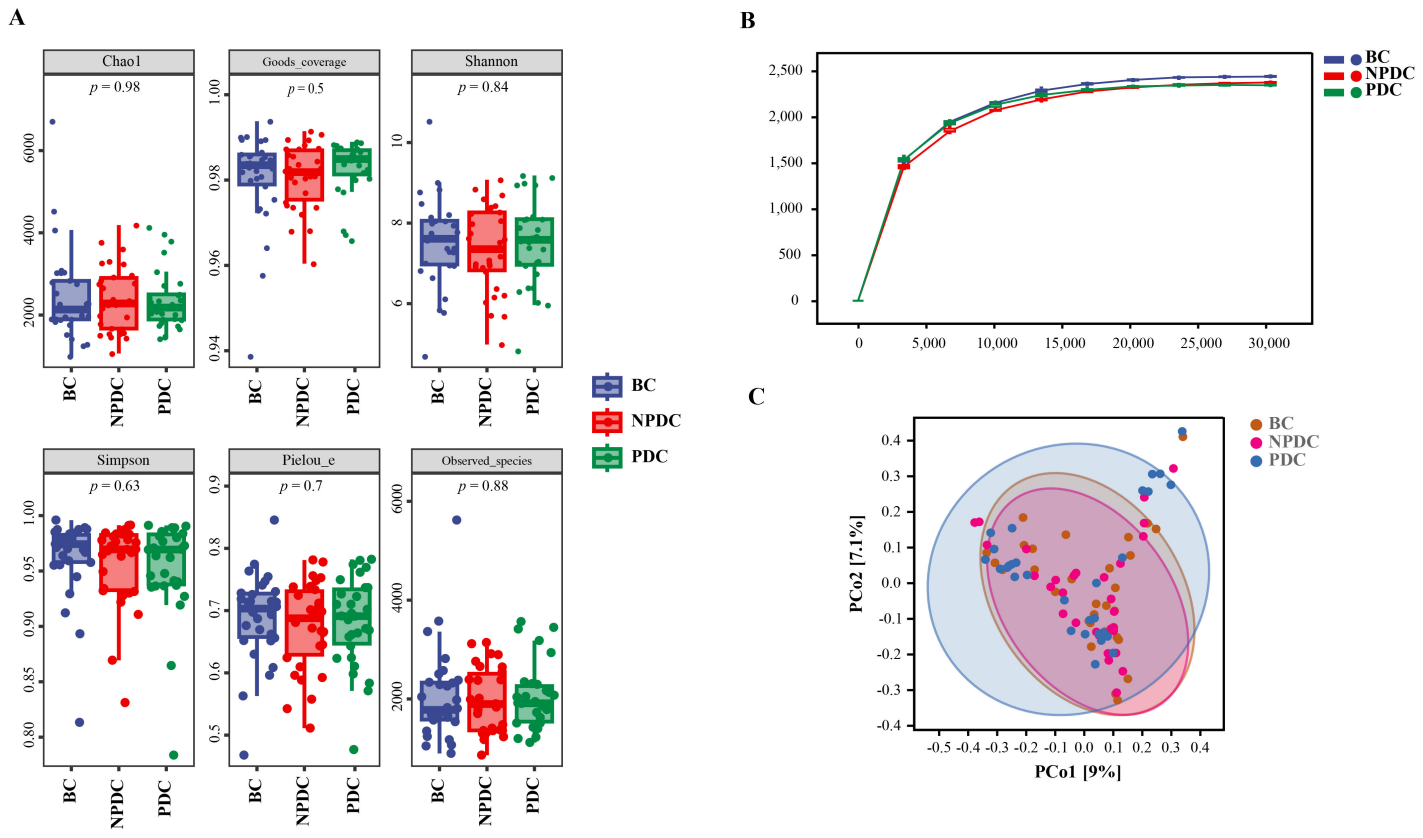
Comparison of intestinal microbial diversity among the NPDC, PDC, and BC populations. **(A)** Box diagram of the α diversity comparison. The horizontal coordinate represents the grouping, the vertical coordinate represents the α diversity index value **(B)** Rarefaction curve. The horizontal coordinate represents the number of sequences obtained by random extraction, and the vertical coordinate represents the number of species under the classification level. The color represents the group. With increasing sequencing depth, the curve tends to flatten out, indicating a reasonable amount of sequencing data. **(C)** PCoA is based on the Bray−Curtis distance algorithm; the dots represent the sample, and the closer the distance is, the more similar the species composition is; in contrast, the greater the difference is, the greater the horizontal and vertical coordinates represent the two characteristic values with the greatest difference among the samples, and the degree of influence is reflected in the form of percentages.

Analysis of the α diversity of the intestinal flora in 90 samples from the 3 groups revealed that the indices (Chao1, observed species, Shannon, Simpson, Good’s coverage, and Pielou’s evenness) were not significantly different among the 3 groups (**Figure 1A**). The species rarefaction curve revealed that the sequencing depth was sufficient, as the species abundances of the NPDC and PDC groups were similar but lower than that of the BC group (**Figure 1B**). PCoA (based on the Bray−Curtis distance) was used to evaluate the changes in the overall community composition. **Figure 1B** shows that there are many overlapping parts among the three groups, and it is difficult to distinguish them clearly (**Figure 1C**). In addition, analysis of similarities (Anosim) based on the Jaccard distance was used to determine whether there were significant differences among the groups. The results revealed that the R values of PDC vs. BC (R= 0.047, *P* = 0.017) or PDC vs. NPDC (R=0.025, *P* = 0.123) were greater than 0, which indicates that the differences between groups were greater than those within groups (**Table 2**). The difference was statistically significant between the BC and PDC groups, whereas the β diversity in the NPDC and BC groups (R=-0.002, *P*=0.494) was not significantly different.

**Table 2:** Analysis of similarities in intestinal microbial diversity among the groups.

Group1	Group 2	Sample size	Permutations	R	*P* value
all	–	90	999	0.023	0.063
BC	NPDC	60	999	-0.002	0.494
BC	PDC	60	999	0.047	0.017
NPDC	PDC	60	999	0.025	0.123

### 3.3 Comparison of the intestinal microbial composition of the NPDC, PDC, and BC populations

**Figure 2:**
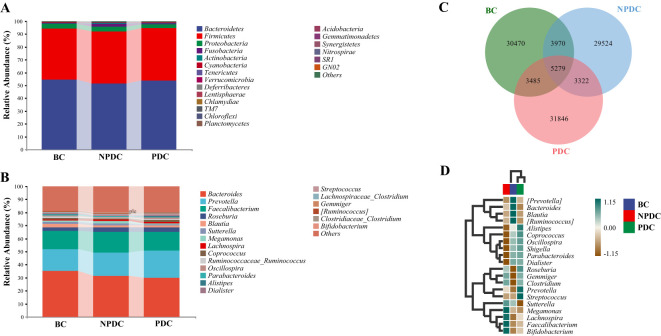
Comparison of the intestinal microbial composition among the NPDC, PDC, and BC populations. The abscissa is the group name, and the ordinate represents the relative abundance of species at the phylum level **(A)** and genus level **(B)**. The bars represent groups. Different colors correspond to different species, and the longer the color block is, the greater the relative abundance of the species at the phylum or genus level. **(C)** Species Venn diagram analysis: the color corresponds to the groups, the overlapping part represents the ASV shared by the three groups, the nonoverlapping part represents the ASV unique to the group, and the number is the corresponding ASV count. **(D)** Species composition heatmaps of the top 20 genera. The horizontal axis represents the group, the right side represents the name of the genus with the top 20 average abundances at the taxonomic level, the left side represents the species clustering tree, and the upper side represents the group clustering tree. The different species abundances are distinguished by the depth of color.

A comparison of the compositions of the intestinal microorganisms revealed that *Bacteroidetes*, *Firmicutes*, *Proteobacteria*, *Fusobacteria*, and *Actinobacteria* were the main dominant bacteria in each group at the phylum level. It accounts for more than 98% of the relative abundance of all bacteria (**Figure 2A**; **Table 3**). *Bacteroidetes* were the main bacteria (BC group: 54.63%, NPDC group: 51.62%, PDC group: 53.93%). The second dominant phylum was *Firmicutes* (BC group: 39.72%, NPDC group: 40.26%, PDC group 40.63%), and the ratio of Firmicutes to Bacteroides (F/B) in the PDC and NPDC groups was greater than that in the BC group. However, these differences were not statistically significant at the phylum level. Next, we analyzed the contributions of the top 20 bacterial genera to each group. As shown in **Figure 2B**; **Table 4**, *Bacteroides* was the most important genus (BC group: 35.29%, NPDC group: 31.31%, PDC group: 30.07%). A Venn diagram can directly reflect the similarity and specificity of species number composition among samples of different groups. The results revealed 5279 ASVs in the three groups, 30,470 ASVs in the BC group, 29,524 ASVs in the NPDC group, and 31,846 ASVs in the PDC group, which were not found in the other two groups (**Figure 2C**). Species composition heatmap analysis at the genus level revealed that, compared with PDC or NPDC subjects with prehypertension, *Bacteroides*, *Blautia*, and [*Ruminococcus*] were enriched in the BC group, and the relative abundance of *Blautia* was significantly different (*P* < 0.05) both in NPDC vs. BC and in PDC vs. BC. *Streptococcus*, *Prevotella*, *Alistipes*, *Coprococcus*, *Oscillospira*, *Parabacteroides*, *and Dialister* were enriched in PDC subjects relative to NPDC subjects. The difference in the abundance of *Alistipes* between PDC and NPDC was statistically significant (*P* < 0.05). These results suggest that the intestinal flora structure of the prehypertensive population is different from that of individuals with ideal blood pressure and that the intestinal flora structures of prehypertensive individuals with PDC and NPDC are also different.

**Table 3:** Average relative abundance of the top 10 species in each group (phylum level).

Phylum	BC (n=30)	NPDC (n=30)	PDC (n=30)	*P* value
*Bacteroidetes*	54.63 **±** 16.03	51.61 **±** 16.85	53.93 **±** 15.71	0.752
*Firmicutes*	39.72 **±** 16.17	40.26 **±** 18.02	40.63 **±** 15.22	0.977
*Proteobacteria*	3.79 **±** 2.07	3.82 **±** 2.28	3.10 **±** 1.98	0.330
*Fusobacteria*	0.36 **±** 1.42	2.40 **±** 8.93	0.85 **±** 4.17	0.363
*Actinobacteria*	0.55 **±** 0.32	0.85 **±** 1.27	0.54 **±** 0.78	0.306
*Cyanobacteria*	0.42 **±** 2.16	0.36 **±** 1.03	0.22 **±** 0.93	0.867
*Tenericutes*	0.05 **±** 0.13	0.06 **±** 0.21	0.12 **±** 0.2	0.300
*Verrucomicrobia*	0.01 **±** 0.02	0.03 **±** 0.06	0.03 **±** 0.06	0.399
*Deferribacteres*	0.01 **±** 0.02	0.02 **±** 0.02	0.01 **±** 0.02	0.359
*Lentisphaerae*	0.01 **±** 0.02	0.01 **±** 0.03	0.02 **±** 0.04	0.435
*Others*	0.46 **±** 0.27	0.58 **±** 0.44	0.56 **±** 0.37	0.357
F/B	36.87 **±** 13.49	43.75 **±** 15.35	44.06 **±** 16.97	0.110

The data are presented as the means ± SDs. No significant difference among the 3 groups.

**Table 4:** Average relative abundance of the top 20 species in each group (genus level).

Genus	BC (n=30)	NPDC (n=30)	PDC (n=30)	*P* value
*Bacteroides*	35.29 **±** 23.06	31.31 **±** 22.96	30.07 **±** 24.86	0.672
*Prevotella*	16.45 **±** 23.75	17.99 **±** 25.91	20.59 **±** 24.98	0.809
*Faecalibacterium*	14.13 **±** 9.21	16.02 **±** 12.5	14.48 **±** 9.85	0.765
*Roseburia*	2.77 **±** 2.08	3.23 **±** 2.90	3.37 **±** 5.22	0.802
*Blautia*	2.59 **±** 2.57	1.42 **±** 1.76^*^	1.37 **±** 1.11^*^	0.024
*Sutterella*	1.50 **±** 1.38	1.63 **±** 1.97	0.94 **±** 0.97	0.172
*Megamonas*	1.00 **±** 2.76	1.75 **±** 6.62	0.88 **±** 4.37	0.754
*Lachnospira*	0.97 **±** 1.52	1.35 **±** 1.68	1.12 **±** 1.06	0.589
*Coprococcus*	1.19 **±** 0.95	0.78 **±** 0.96	1.33 **±** 1.85	0.246
*Ruminococcaceae_Ruminococcus*	0.82 **±** 1.11	0.60 **±** 0.70	0.93 **±** 1.01	0.398
*Oscillospira*	0.87 **±** 1.13	0.54 **±** 0.64	0.93 **±** 0.84	0.203
*Parabacteroides*	0.64 **±** 0.53	0.49 **±** 0.76	0.64 **±** 0.55	0.572
*Alistipes*	0.58 **±** 0.73	0.33 **±** 0.39^#^	0.79 **±** 1.02^^^	0.075
*Dialister*	0.61 **±** 1.13	0.38 **±** 0.44	0.60 **±** 0.84	0.498
*Streptococcus*	0.45 **±** 0.21	0.45 **±** 0.23	0.62 **±** 0.99	0.451
*Lachnospiraceae_Clostridium*	0.59 **±** 0.78	0.43 **±** 0.44	0.40 **±** 0.31	0.345
*Gemmiger*	0.35 **±** 0.31	0.43 **±** 0.57	0.43 **±** 0.45	0.754
*[Ruminococcus]*	0.59 **±** 1.67	0.31 **±** 0.73	0.27 **±** 0.51	0.462
*Clostridiaceae_Clostridium*	0.14 **±** 0.16	0.49 **±** 1.53	0.47 **±** 0.86	0.330
*Bifidobacterium*	0.19 **±** 0.26	0.48 **±** 1.25	0.27 **±** 0.68	0.400
*Others*	18.27 **±** 7.80	19.58 **±** 11.32	19.52 **±** 9.33	0.836

The data are presented as the means ± SDs. Compared with the BC group, ^*^ *P* < 0.05; compared with the NPDC group, ^#^ *P* < 0.05; compared with the PDC group, ^^^ *P* < 0.05.

### 3.4 Analysis of significant differences among the groups

**Figure 3:**
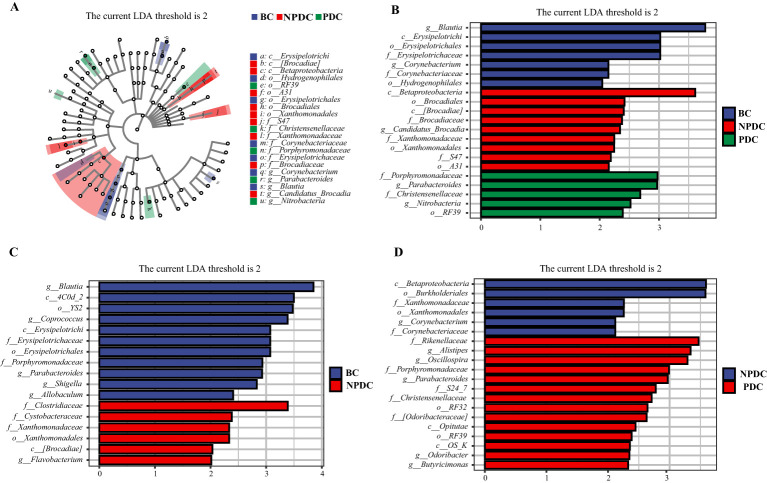
Analysis of the significant differences in LEfSe among all groups (*P <*0.05, LDA>2). **(A)** Cladogram plot. The inner to outer rings represent taxonomic levels from phylum to genus. Each circle at a different classification level represents a classification at that level, and the size of the circle diameter is proportional to the relative abundance. The colors correspond to the groups, and the nodes on the ring represent the microbial groups that play important roles in the corresponding groups. **(B)** LDA histogram: color blocks correspond to groups, and the length is proportional to the LDA score. The higher the LDA score is, the greater the contribution of the species abundance to the difference between groups; **(C)** NPBS vs. BC, **(D)** PDC. vs. NPDC.

To further identify species with significant differences between groups, we performed linear discriminant analysis (LDA) and effect size (LEfSe) analysis (LDA =2). While performing differential analysis directly for all taxonomic levels simultaneously, more emphasis is placed on finding robust differential species between groups. Three classes, six orders, seven families, and five genera met the conditions that the LDA was greater than 2, and no phyla met the conditions (**Table 5**, **Figure 3A**). The relative abundances of *o_RF39*, *f_Porphyromonadaceae*, *f_Christensenellaceae*, *g_parabacteroides*, and *g_nitrobacteria* were significantly greater in the population of the PDC group than in either the NPDC or BC group. (**Table 5**; **Figure 3B**).

**Table 5:** Species differences among the three groups at the class, order, family, and genus levels.

Taxa	Abundance	Group	LDA_score	*P* value
Class Level				
*c_Betaproteobacteria*	4.349	NPDC	3.61	0.049
*c_Erysipelotrichi*	3.633	BC	3.02	0.035
*c_Brocadiae*	1.875	NPDC	2.41	0.016
Order level				
*o_Erysipelotrichales*	3.633	BC	3.02	0.035
*o_Brocadiales*	1.875	NPDC	2.42	0.016
*o_RF39*	2.814	PDC	2.39	0.020
*o_Xanthomonadales*	2.889	NPDC	2.25	0.035
*o_A31*	1.245	NPDC	2.15	0.047
*o_Hydrogenophilales*	1.350	BC	2.05	0.035
Family level				
*f_Erysipelotrichaceae*	3.633	BC	3.02	0.035
*f_Porphyromonadaceae*	3.839	PDC	2.98	0.030
*f_Christensenellaceae*	3.211	PDC	2.68	0.042
*f_Brocadiaceae*	1.875	NPDC	2.38	0.016
*f_Xanthomonadaceae*	2.889	NPDC	2.25	0.032
*f_S47*	1.245	NPDC	2.19	0.047
*f_Corynebacteriaceae*	2.702	BC	2.15	0.012
Genus level				
*g_Blautia*	4.413	BC	3.78	0.0498
*g_Parabacteroides*	3.809	PDC	2.97	0.037
*g_Nitrobacteria*	0.783	PDC	2.52	0.047
*g_Candidatus_Brocadia*	1.875	NPDC	2.34	0.016
*g_Corynebacterium*	2.702	BC	2.15	0.012

Accordingly, we analyzed the differences in the fecal microbiota between PDC vs.NPDC, NPDC vs. BC, and PDC vs. BC at the genus level and selected bacteria for which *P* < 0.05 and LDA > 2 (**Table 6**). Compared with those in the BC group, the abundances of four species of bacteria in the PDC group significantly changed, and the abundances of *Actinomyces*, *Allobaculum*, *Corynebacterium*, and *Sutterella* decreased in the PDC group (**Table 6**). Compared with those in the BC group, the abundance of *Flavobacterium* in the NPDC group was significantly greater, and the abundances of *Allobaculum*, *Blautia*, *Coprococcus*, *Parabacteroides*, and *Shigella* were significantly lower (**Figure 3C**), suggesting that these bacterial genera are specific to prehypertension. The differences between the NPDC and BC groups reflect the distinction between prehypertension and ideal blood pressure. To further clarify the effects of WC and BMI on the differentiation of the NPDC and BC groups, we performed univariate and multivariate logistic regression analyses, respectively. Univariate logistic regression analyses revealed that the ORs of WC and BMI were 1.114 (95% CI: 1.041–1.193, *P* =0.002) and 1.273 (95% CI: 1.047–1.548, *P*=0.015), respectively, suggesting that both BMI and WC are risk factors for prehypertension. Through multifactorial logistic regression analysis, BC and BMI were corrected, and WC was included in the regression equation (OR=1.128, 95% CI: 1.016–1.253, *P*=0.024), suggesting that WC was an independent risk factor for prehypertension. The taxa with significant differences between the PDC group and the NPDC group were related to the phlegm dampness constitution of prehypertension. As shown in **Figure 3D**, a total of six bacterial genera presented significantly different abundances between PDC and NPDC. Among them, *Alistipes*, *Butyricimonas*, *Odoribacter*, *Oscillospira*, and *Parabacteroides* were enriched in the PDC group, and *Corynebacterium* was enriched in the NPDC group.

**Table 6:** Pairwise comparison of bacterial genera with significant differences between groups.

Genus	Abundance	Group	LDA_score	*P* value
PDC vs. NPDC				
*Alistipes*	3.895	PDC	3.34	0.031
*Butyricimonas*	2.989	PDC	2.33	0.041
*Corynebacterium*	2.655	NPDC	2.12	0.013
*Odoribacter*	2.965	PDC	2.35	0.019
*Oscillospira*	3.967	PDC	3.29	0.030
*Parabacteroides*	3.809	PDC	2.97	0.020
PDC vs. BC				
*Actinomyces*	2.864	BC	2.04	0.030
*Allobaculum*	2.931	BC	2.41	0.049
*Corynebacterium*	2.702	BC	2.16	0.008
*Sutterella*	4.176	BC	3.43	0.043
NPDC vs. BC				
*Allobaculum*	2.931	BC	2.41	0.037
*Blautia*	4.413	BC	3.85	0.027
*Coprococcus*	4.076	BC	3.39	0.049
*Flavobacterium*	1.406	NPDC	2.01	0.003
*Parabacteroides*	3.803	BC	2.93	0.035
*Shigella*	3.425	BC	2.83	0.027

### 3.5 Specific intestinal microbial screening and diagnostic capabilities of prehypertensive PDCs

**Figure 4:**
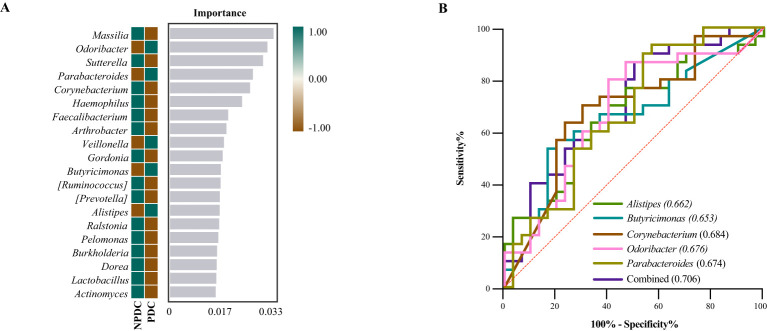
Screening of the differential intestinal flora of prehypertensive PDC and establishment of a diagnostic model. **(A)** Random forest analysis. Heatmaps showing the abundance distributions of these bacterial genera in both groups, which decreased in importance to the model from top to bottom. **(B)** ROC analysis of different strains and their combined diagnostic models between PDC and NPDC.

To further screen the specific flora of PDC in the prehypertensive population, random forest analysis was performed between the PDC group and NPDC group via LEfSe analysis. The results revealed that *Alistipes*, *Butyricimonas*, *Odoribacter*, *Parabacteroides*, and *Corynebacterium* were the bacterial genera whose abundances significantly differed between the NPDC and PDC and strongly contributed to group differentiation (**Figure 4A**; **Table 7**). We noted that these five bacteria are also genera with significant intergroup differences in NPDC vs. PDC according to LEfse analysis (**Table 6**). ROC analysis was used to further explore the potential of these 5 strains in the identification of phlegm dampness in the prehypertensive population. AUC, sensitivity, specificity, and threshold values were used to assess diagnostic and predictive accuracy (**Table 8**). The AUC of the PDC compared with the NPDC ranged from 0.653 (95% CI: 0.511–0.794) to 0.684 (95% CI 0.547–0.822) (**Table 8**; **Figure 4B**). We further constructed a combined diagnostic model of these 5 bacterial genera via logistic regression, and the results revealed that the model could better distinguish PDC from NPDC, with an AUC of 0.706 (95% CI: 0.573–0.838) (**Table 8**; **Figure 4B**).

**Table 7:** Random forest importance analysis of the top 20 bacterial genera in PDC vs. NPDC.

Genus	NPDC	PDC
*Massilia*	17.43	7.20
*Odoribacter*	23.20	39.53
*Sutterella*	858.53	446.50
*Parabacteroides*	250.63	296.80
*Corynebacterium*	24.70	9.23
*Haemophilus*	131.80	121.77
*Faecalibacterium*	9245.90	6777.20
*Arthrobacter*	12.97	8.03
*Veillonella*	65.27	72.77
*Gordonia*	8.1	2.53
*Butyricimonas*	26.87	42.90
*[Ruminococcus]*	157.73	113.53
*[Prevotella]*	243.07	156.10
*Alistipes*	169.97	330.60
*Ralstonia*	20.93	17.33
*Pelomonas*	5.1	2.07
*Burkholderia*	74.1	59.53
*Dorea*	93.83	76.53
*Lactobacillus*	111.40	103.03
*Actinomyces*	34.07	24.63

**Table 8:** Diagnostic test evaluation indices of the model.

Genus	Sensitivity %	Specificity %	*Yorden Index*	AUC	95% CI	*P* value
*Alistipes*	76.67	53.33	0.300	0.662	0.523 to 0.801	0.031
*Butyricimonas*	56.67	80.00	0.367	0.653	0.511 to 0.794	0.042
*Corynebacterium*	70.00	70.00	0.400	0.684	0.547 to 0.822	0.014
*Odoribacter*	86.67	53.33	0.400	0.676	0.537 to 0.816	0.019
*Parabacteroides*	90.00	46.67	0.367	0.674	0.537 to 0.812	0.020
Combined	90.00	46.67	0.367	0.706	0.573 to 0.838	0.006

## 4 Discussion

Compared with other constitutions, the PDC is more closely associated with metabolic diseases (Ma et al., 2017; Shen et al., 2019; Zhou and Wang, 2019; Zhu et al., 2021; Linghui et al., 2024) and is intimately related to hypertension and prehypertension (Qian et al., 2003; Wang, 2015; Zhu et al., 2021). The PDC population has been reported to present aberrant expression via genomics (Wang et al., 2013; Yao et al., 2018; Dong et al., 2021), proteomics (Li et al., 2022; Linghui et al., 2024), and metabolomics (You et al., 2024). Metabolic disorders occurring in individuals with PDC are associated with small intestine dysfunction (Zhang et al., 2023). In turn, structural changes and disproportionate proportions of intestinal microorganisms are prominent features of metabolic disorders (Pedersen et al., 2016). Therefore, phlegm dampness and metabolic disorders are particularly closely related to the gut microbiome, and the gut microbiome provides a new direction and breakthrough for the research of PDC and metabolic disorders such as hypertension (Liang et al., 2020).

In this study, we examined the gut microbiota characteristics of individuals with prehypertension and prehypertension combined with PDC. The species abundances of the NPDC and PDC groups were very similar; both were lower than that of the BC group, although the difference was not statistically significant. In terms of β diversity, only the PDC and BC groups presented significant differences. The Venn diagram based on the ASV level and species composition heatmap analysis at the genus level suggested that the intestinal flora structure of the prehypertensive population might differ from that of the ideal blood pressure. This finding is consistent with the study of Li et al (Li et al., 2017). In addition, [*Prevotella*] and *Bacteroides* tended to increase or decrease sequentially among the BC, NPDC, and PDC groups, indicating that NPDC seems to be the intermediate state between the BC and PDC groups.

Research involving 61,475 participants revealed that BMI and WC were more strongly associated with prehypertension than any other index of obesity was, with a significantly greater AUC for WC (Xiao et al., 2023). A longitudinal genomic and epidemiologic-based study reported that BMI and WC were identified as important risk factors for hypertension (Jang et al., 2024). Our results revealed significant differences in WC and BMI between the NPDC and BC groups and were risk factors for prehypertension, which is consistent with the findings of previous studies. These findings suggest that metabolic disorders are already present in prehypertensive populations and may have an impact on the structure and composition of the intestinal flora. Our results revealed that the relative abundance of *Blautia* was significantly lower in the NPDC and PDC groups, which were both in the prehypertensive stage, than in the BC group, which was ideally pressurized. Similarly, previous studies have reported an inverse correlation between *Blautia* and systolic blood pressure (Toral et al., 2019), with a lower relative abundance in hypertensive patients (Chang et al., 2020). In a study of 377 subjects, Mizoguchi R et al (Mizoguchi et al., 2023). reported that *Blautia* was associated with parameters of the renin-angiotensin-aldosterone system (RAAS), which is considered to be a regulatory mechanism of the endocrine system associated with hypertension.

We focused on the intestinal flora, which differed significantly between PDC and NPDC because they may be the key characteristic flora of the prehypertensive PDC population. In our study, neither blood pressure nor laboratory tests differed significantly between the PDC and NPDC groups, which somewhat excludes the influence of potential confounders such as weight, WC, BMI, lipids, and blood glucose on gut flora characteristics in distinguishing between PDC and NPDC. Our results indicated that the intestinal flora structures of PDC and NPDC differed. To further explore the intestinal flora characteristics of PDC in prehypertensive individuals, in addition to LEfSe analysis, random forest analysis was also carried out between PDC and NPDC. The results revealed that *Alistipes*, *Butyricimonas*, *Odoribacter*, *Parabacteroides*, and *Corynebacterium* were the bacterial genera whose abundances significantly differed between PDC and NPDC and strongly contributed to group differentiation.

*Alistipes* is a relatively new genus of bacteria that has been isolated mainly from medical clinical samples. From the perspective of ecology, *Alistipes* species exist mainly in the gut of healthy humans (Shkoporov et al., 2015), while studies have reported that they may have novel effects on inflammation, cancer, and mental health (Parker et al., 2020). A positive correlation between the abundance of *Alistipes* and SBP or DBP in untreated hypertensive patients has been reported (Li et al., 2019). Deng et al. (Huan et al., 2025) applied the active peptide KYPHVF (KF6) to control hypertension by inhibiting ACE activity and reported that KF6 reduced the abundance of *Alistipes* to regulate the composition of the gut microbiota. In our study, *Alistipes* was significantly enriched in the PDC group relative to the NPDC group (*p* < 0.05). Dong et al (Dong et al., 2022). indicated that ACEIs/ARBs improved the gut microbial composition and function in HTN patients by reducing potential pathogenic bacteria and increasing beneficial bacteria such as *Odoribacter*. Angoorani P et al. (Angoorani et al., 2021) indicated that *Odoribacter* abundance increased significantly during intermittent fasting, which had positive effects on hypertension. In addition, animal studies have shown that the diameter of abdominal aortic aneurysms, which are closely associated with hypertension, correlates with *Odoribacter* and is important for the progression of the disease (Xie et al., 2020). However, few studies have reported whether *Odoribacter* is associated with prehypertension or phlegm dampness, and our study fills this gap.

Macrogenomic sequencing analysis revealed that *Parabacteroides*, an opportunistic pathogen, is frequently distributed in the intestines of hypertensive patients and has the potential to predict hypertension (Yan et al., 2017). Researchers have reported that six bacterial genera, including *Parabacteroides*, are associated with obese patients suffering from various metabolic disorders (Zeng et al., 2019). Our results revealed a significantly greater abundance of *Parabacteroides* in the PDC group than in the NPDC group. In addition, we found that *Corynebacterium* was enriched in the NPDC group but not in the PDC group. Gomez-Arango et al. (Gomez-Arango et al., 2016) suggested that *Corynebacterium* abundance was negatively correlated with systolic blood pressure, which may be related to reduced butyrate production and increased plasminogen activator inhibitor-1 concentration. Wu YJ et al. (Wu et al., 2018) reported that *Corynebacterium* spp. were lower in the obese group than in the healthy population through characterization of the salivary microbiome. *Alistipes*, *Butyricimonas*, *Odoribacter*, and *Parabacteroides, which are* enriched in PDC, belong to the *Bacteroides* phylum, whereas *Corynebacterium*, which belongs to *the Actinobacteria* phylum, was exhausted in PDC. The gut flora may change with the same trend along with changes in body constitution. In contrast, the PDC and NPDC groups did not significantly differ in traditional clinical indicators. Microbiological characteristics can be utilized as biomarkers to differentiate between prehypertensive populations with or without a PDC, which provides data support and evidence for research on phlegm-dampness constitution in prehypertensive individuals and has good application prospects.

There are several limitations to our study. This is a single-center case−control study with a larger sample size, and multicenter external validation will more strongly support our results. In addition, 16S rRNA sequencing may have certain limitations in the functional study of the intestinal flora, and metagenomic sequencing is helpful for better research on the function of the intestinal flora.

## 5 Conclusion

In summary, our study suggests that the gut microbiota composition is different between PDC individuals and NPDC subjects with prehypertension. The specific bacterial characteristics show potential diagnostic value in distinguishing prehypertensive individuals with phlegm dampness. These findings improve the study of prehypertension from the perspective of traditional Chinese medicine and provide potential targets and new perspectives for the study of prehypertension, which may contribute to the prevention and treatment of prehypertension more accurately.

## Data Availability

The data analyzed in this study was available from the NCBI database (https://www.ncbi.nlm.nih.gov/sra/PRJNA1220193).
