# Supplementary material for: Investigating the Gut Microbiota Profile in Prehypertensive Individuals Exhibiting Phlegm-Dampness Constitution
Source: Front Cell Infect Microbiol. 2025 Mar 4;15:1507076. doi: 10.3389/fcimb.2025.1507076 (PMC11913815; doi:10.3389/fcimb.2025.1507076)
Supplement: Supplementary file 1 [file Table1.docx]

**Supplementary Table**

**TCM Constitution Classification Questionnaire**

| **Experience/condition in the past year** | **No** | **Slightly** | **Sometimes** | **Often** | **All the time** |
| --- | --- | --- | --- | --- | --- |
| (1) Were you energetic? | 1 | 2 | 3 | 4 | 5 |
| (2) Did you get tired easily? | 1 | 2 | 3 | 4 | 5 |
| (3) Did you experience shortness of breath? | 1 | 2 | 3 | 4 | 5 |
| (4) Did you get palpitations? | 1 | 2 | 3 | 4 | 5 |
| (5) Did you get dizzy easily or become dizzy when standing up? | 1 | 2 | 3 | 4 | 5 |
| (6) Did you prefer quietness and not like to talk? | 1 | 2 | 3 | 4 | 5 |
| (7) Was your voice weak when talking? | 1 | 2 | 3 | 4 | 5 |
| (8) Did you feel in low spirits and depressed? | 1 | 2 | 3 | 4 | 5 |
| (9) Did you easily feel anxious and worried? | 1 | 2 | 3 | 4 | 5 |
| (10) Did you feel overly sensitive, vulnerable, or emotionally upset? | 1 | 2 | 3 | 4 | 5 |
| (11) Were you easily scared or frightened? | 1 | 2 | 3 | 4 | 5 |
| (12) Did you experience distention in the underarms or breasts? | 1 | 2 | 3 | 4 | 5 |
| (13) Did you feel chest or abdominal stuffiness? | 1 | 2 | 3 | 4 | 5 |
| (14) Did you sigh without reason? | 1 | 2 | 3 | 4 | 5 |
| (15) Did your body feel heavy or lethargic? | 1 | 2 | 3 | 4 | 5 |
| (16) Did the palms of your hands or soles of your feet feel hot? | 1 | 2 | 3 | 4 | 5 |
| (17) Did your hands or feet feel cold or clammy? | 1 | 2 | 3 | 4 | 5 |
| (18) Did you feel cold easily in your abdomen, back, lower back, or knees? | 1 | 2 | 3 | 4 | 5 |
| (19) Were you sensitive to cold and tended to wear more clothes than others? | 1 | 2 | 3 | 4 | 5 |
| (20) Did your body and face feel hot? | 1 | 2 | 3 | 4 | 5 |
| (21) Did you feel more vulnerable to the cold than others (winter coldness, air conditioners, fans, etc.)? | 1 | 2 | 3 | 4 | 5 |
| (22) Did you catch colds more easily than others? | 1 | 2 | 3 | 4 | 5 |
| (23) Did you sneeze even when you did not have a cold? | 1 | 2 | 3 | 4 | 5 |
| (24) Did you have a runny or stuffy nose even when you did not have a cold? | 1 | 2 | 3 | 4 | 5 |
| (25) Did you cough due to seasonal changes, temperature changes, or unpleasant odors? | 1 | 2 | 3 | 4 | 5 |
| (26) Did you sweat easily when your physical activity increased slightly? | 1 | 2 | 3 | 4 | 5 |
| (27) Did you forget things easily? | 1 | 2 | 3 | 4 | 5 |
| (28) Did you have an excessively oily forehead and/or T-zone? | 1 | 2 | 3 | 4 | 5 |
| (29) Were your lips redder than in the past? | 1 | 2 | 3 | 4 | 5 |
| (30) Did you have allergies? (e.g. medicine, food, odors, pollen, pet dander, or during seasonal or weather changes, etc.) Experience/condition in the past year No Slightly Sometimes Often All the time | 1 | 2 | 3 | 4 | 5 |
| (31) Did you get hives/urticaria easily? | 1 | 2 | 3 | 4 | 5 |
| (32) Did your skin have purpura (purple spots, ecchymosis) due to allergies? | 1 | 2 | 3 | 4 | 5 |
| (33) Did black or purple bruises appear on your skin for no reason? | 1 | 2 | 3 | 4 | 5 |
| (34) Did your skin turn red and show traces when you scratched it? | 1 | 2 | 3 | 4 | 5 |
| (35) Did your skin or lips feel dry? | 1 | 2 | 3 | 4 | 5 |
| (36) Did you have visible capillary (thread) veins on your cheeks? | 1 | 2 | 3 | 4 | 5 |
| (37) Did you feel pain somewhere in your body? | 1 | 2 | 3 | 4 | 5 |
| (38) Did you experience hot flashes? | 1 | 2 | 3 | 4 | 5 |
| (39) Did your nose or your face feel greasy, oily, or shiny? | 1 | 2 | 3 | 4 | 5 |
| (40) Did you have a dark face or get brown spots easily? | 1 | 2 | 3 | 4 | 5 |
| (41) Did you get acne or sores easily? | 1 | 2 | 3 | 4 | 5 |
| (42) Did you have upper eyelid swelling? | 1 | 2 | 3 | 4 | 5 |
| (43) Did you get dark circles under the eyes easily? | 1 | 2 | 3 | 4 | 5 |
| (44) Did your eyes feel dry and you used eye drops? | 1 | 2 | 3 | 4 | 5 |
| (45) Were your lips darker, more blue or purple than usual? | 1 | 2 | 3 | 4 | 5 |
| (46) Did you often feel parched and need to drink water? | 1 | 2 | 3 | 4 | 5 |
| (47) Did your throat feel strange (i.e., as if something was stuck or there was a lump in your throat)? | 1 | 2 | 3 | 4 | 5 |
| (48) Did you have a bitter or strange taste in your mouth? | 1 | 2 | 3 | 4 | 5 |
| (49) Did your mouth feel sticky? | 1 | 2 | 3 | 4 | 5 |
| (50) Was your abdomen flabby? | 1 | 2 | 3 | 4 | 5 |
| (51) Did you have an abundance of phlegm, especially in your throat? | 1 | 2 | 3 | 4 | 5 |
| (52) Did you feel uncomfortable when you drank or ate something cold, or did you avoid drinking or eating cold items? | 1 | 2 | 3 | 4 | 5 |
| (53) Could you adapt yourself to external natural or social environment changes? | 1 | 2 | 3 | 4 | 5 |
| (54) Did you easily experience insomnia? | 1 | 2 | 3 | 4 | 5 |
| (55) Did you easily contract diarrhea when you were exposed to cold or ate (or drank) something cold? | 1 | 2 | 3 | 4 | 5 |
| (56) Did you pass sticky stools and/or feel that your bowel movement was incomplete? | 1 | 2 | 3 | 4 | 5 |
| (57) Did you get constipated easily or have dry stools? | 1 | 2 | 3 | 4 | 5 |
| (58) Did your tongue have a thick coating? | 1 | 2 | 3 | 4 | 5 |
| (59) Did your urethral canal feel hot when you urinated, or did your urine have a dark color? | 1 | 2 | 3 | 4 | 5 |
| (60) Was your vaginal discharge yellowish (only for female interviewees)? | 1 | 2 | 3 | 4 | 5 |
| (60) Was your scrotum always wet (only for male interviewees)? | 1 | 2 | 3 | 4 | 5 |

This questionnaire aims to survey your constitution and subsequently provide a reference for future health management and clinical diagnosis. Please read every question carefully and choose the most suitable response on the basis of your actual situation or experience in the past year. If you are unsure of the answer to a specific question, choose the answer that is most similar to your actual situation. Make sure that you answer all the questions on the basis of your situation in the past year (excluding the effect of drugs) and give only one answer to each question. Experience/conditions in the past year: No, Slightly, Sometimes, Often, All the time.

【No】Never happened in the past year.

【Slightly】Occasionally occurred in the past year.

【Sometimes】This occurred, but there was no regular pattern.

【Often】It has occurred most of the time in the past year.

【All the time】It happened all the time in the past year.

Original scores: Sum each item’s score

Converted scores: [(original scores - items)/(items*4)] * 100

**Determination:**

| Constitution | Condition | Result |
| --- | --- | --- |
| Balanced constitution | Converted scores>60 | Yes |
|  | The rest constitutions’ converted scores all<30 |  |
|  | Converted scores>60 | Basically yes |
|  | The rest constitutions’ converted scores all<40 |  |
|  | Not stratified the conditions above | No |
| Unbalanced constitution | Converted scores>40 | Yes |
|  | Converted scores39~30 | Tend to |
|  | Converted scores<30 | No |

**Balanced Constitution**

Original scores = item 2 + item 7 + item 21 + item 27 + item 8 + item 1 + item 53 + item 54

Converted scores = [((item 2 + item 7 + item 21+ item 27 + item 8 + item 1 + item 53 + item 54)-8)/8*4] *100

**Phlegm dampness Constitution**

Original scores = item 13 + item 15 + item 50 + item 28 + item 42 + item 49 + item 51 + item 58

Converted scores = [((item 13 + item 15 + item 50 + item 28 + item 42 + item 49 + item 51 + item 58)-8)/8*4] *100
